# Ursolic Acid Attenuates Hepatic Steatosis, Fibrosis, and Insulin Resistance by Modulating the Circadian Rhythm Pathway in Diet-Induced Obese Mice

**DOI:** 10.3390/nu10111719

**Published:** 2018-11-09

**Authors:** Eun-Young Kwon, Su-Kyung Shin, Myung-Sook Choi

**Affiliations:** 1 Department of Food Science and Nutrition, Kyungpook National University, 1370 San-Kyuk Dong Puk-Ku, Daegu 41566, Korea; savage20@naver.com; 2 Center for Food and Nutritional Genomics Research, Kyungpook National University, 1370 San-Kyuk Dong Puk-Ku, Daegu 41566, Korea; 3 Department of Physiology & Obesity-Mediated Disease Research Center, Keimyung University School of Medicine, Daegu 82601, Korea; ssk1210@hanmail.net

**Keywords:** fibrosis, extracellular matrix, ursolic acid, circadian rhythm, liver-specific

## Abstract

The aim of the current study was to elucidate the effects of long-term supplementation with dietary ursolic acid (UR) on obesity and associated comorbidities by analyzing transcriptional and metabolic responses, focusing on the role of UR in the modulation of the circadian rhythm pathway in particular. C57BL/6J mice were divided into three groups and fed a normal diet, high-fat diet, or high-fat + 0.05% (*w*/*w*) UR diet for 16 weeks. Oligonucleotide microarray profiling revealed that UR is an effective regulator of the liver transcriptome, and canonical pathways associated with the “circadian rhythm” and “extracellular matrix (ECM)–receptor interactions” were effectively regulated by UR in the liver. UR altered the expression of various clock and clock-controlled genes (CCGs), which may be linked to the improvement of hepatic steatosis and fibrosis via lipid metabolism control and detoxification enhancement. UR reduced excessive reactive oxygen species production, adipokine/cytokine dysregulation, and ECM accumulation in the liver, which also contributed to improve hepatic lipotoxicity and fibrosis. Moreover, UR improved pancreatic islet dysfunction, and suppressed hepatic gluconeogenesis, thereby reducing obesity-associated insulin resistance. Therapeutic approaches targeting hepatic circadian clock and CCGs using UR may ameliorate the deleterious effects of diet-induced obesity and associated complications such as hepatic fibrosis.

## 1. Introduction

Obesity represents one of the most severe burdens on healthcare systems, and is closely related to the increased risk of multiple chronic conditions, including insulin resistance (IR), diabetes, cardiovascular diseases, fatty liver, fibrosis, non-alcoholic steatohepatitis, and other comorbidities [1]. Molecular links between obesity, hepatic steatosis, and liver fibrosis remain incompletely understood but may include chronic inflammation and extracellular matrix (ECM) accumulation in the liver. One hypothesis explaining the link between obesity, hepatic steatosis, and liver fibrosis is the “two-hit hypothesis”, which implies that the accumulation of lipid metabolites can lead to a series of events including lipotoxicity, oxidative stress, and inflammation that produce a “second hit” [2]. Another hypothesis suggests that the “second hit” promotes tissue injury and the activation of stellate cells. Activated stellate cells are recognized as the primary cellular source of matrix components in the liver, and play a central role in the development of hepatic fibrosis by accumulating ECM proteins in the space of Disse via accelerated pro-inflammatory responses [3,4]. Moreover, genome-wide gene expression studies provided the first and important insights into the role of the circadian clock in liver metabolism, and suggest that the interaction between altered energy metabolism and disruptions in the circadian clock results in a downward spiral that can cause obesity, IR, hepatic steatosis, and other metabolic diseases or exacerbate pathological states [5,6,7,8,9], though the underlying mechanisms remain largely unknown.

Ursolic acid (UR), or 3β-hydroxy-urs-12-en-28-oic acid, is a pentacyclic triterpenoid found in large amounts in berries, leaves, flowers, and fruits of medicinal plants, such as *Rosmarinus officinalis*, *Eriobotrya japonica*, and *Eugenia jambolana* [10,11,12,13,14,15]. UR exerts anti-inflammatory, anti-oxidative, anti-cancer, anti-obesity, and anti-diabetic activities by activating peroxisome proliferator-activated receptor alpha (PPAR-α) and 5′ AMP-activated protein kinase (AMPK) and increasing energy expenditure. However, the effects of this chemical on hepatic fibrosis are not fully elucidated, and to date, few studies have been conducted to investigate the effects of long-term treatment with UR based on the integration of transcriptional profiles and phenotypic biomarkers. Transcriptomic studies are a common approach for the identification of molecular mechanisms underlying the anti-metabolic disease effects of traditional medicines. In the current study, we evaluated the effects of long-term supplementation with dietary UR and the molecular mechanisms underlying its metabolic regulatory effects in diet-induced obesity (DIO) and metabolic disease using transcriptomic analysis. This study demonstrates for the first time that dietary UR attenuated inflammation-mediated metabolic diseases including DIO, hepatic steatosis, dyslipidemia, fibrosis, and IR by controlling the circadian clock and suppressing hepatic ECM remodeling in DIO mice.

## 2. Materials and Methods

### 2.1. Animals

Thirty-six male C57BL/6J mice were obtained from the Jackson Laboratory (Bar Harbor, ME, USA) at 4 weeks of age. Mice were individually housed under a constant temperature (24 °C) and 12-h light/dark cycles, fed an AIN-76 semi-purified diet for a 1-week acclimation period, and then randomly divided into three groups. The mice were fed a normal diet (ND, *n* = 10), high-fat diet (HFD) consisting of 20% fat and 1% cholesterol (*n* = 13), or HFD with 0.05% (*w*/*w*) UR (*n* = 13; UR obtained from Sigma-Aldrich, St. Louis, MO, USA) for 16 weeks. All experimental diets were prepared weekly and stored in a dark room at 4 °C. Body weights (BW) and blood glucose levels were recorded every 2 weeks. At the end of the experimental period, all mice were anesthetized with ether after a 12-h fast. Blood was collected from the inferior vena cava to determine the glucose, plasma lipid, and hormone concentrations. Tissues were removed, rinsed with physiological saline, weighed, immediately frozen in liquid nitrogen, and stored at −70 °C until analysis. The animal study protocols were approved by the Ethics Committee at Kyungpook National University (approval no. KNU 2010-4-14).

### 2.2. Blood Glucose, Intraperitoneal Glucose Tolerance Test (IPGTT), Homeostasis Model Assessment-Estimated IR (HOMA-IR) Index, and Plasma Biomarkers

Every 2 weeks, 12-h fasting blood glucose levels were measured in the tail vein using a glucose analyzer (Lifescan Inc., Wayne, PA, USA). For the IPGTT, 5 weeks after the start of the experiment, 12-h-fasted mice were injected intraperitoneally with glucose (0.5 g/kg BW), and blood glucose levels were measured in the tail vein after 0, 30, 60, and 120 min. Radioimmunometric assays were used to measure plasma insulin and glucagon concentrations using a multiplex detection kit (Bio-Rad, Millipore, Hercules, CA, USA). The HOMA-IR index was calculated according to the following formula: (fasting insulin concentration (mU/L) × fasting glucose concentration (mg/dL) × 0.05551)/22.5.

### 2.3. Analyses of Plasma and Hepatic Lipids

The levels of total plasma cholesterol (TC), high-density lipoprotein cholesterol (HDL-C), triglycerides (TG), glutamic oxaloacetic transaminase (GOT), glutamic pyruvic transaminase (GPT), and phospholipids were measured using enzymatic assay kits (Asan Pharm Co., Seoul, Korea) as were those of plasma free fatty acid (FFA) (Wako Chemicals, Osaka, Japan), apolipoprotein A1 (apoA1; Eiken Chemical Co., Tokyo, Japan), and apolipoprotein B100 (apoB100; Eiken Chemical Co.). The non-HDL-C value, HDL-C-to-TC ratio (HTR), and atherogenic index (AI) were calculated as follows: non-HDL-C = TC − HDL-C − (TG/5); HTR (%) = HDL-C/TC × 100; and AI = (TC − HDL-C)/HDL-C. Concentrations of plasma adiponectin, leptin, tumor necrosis factor-α (TNF-α), monocyte chemoattractant protein-1 (MCP-1), and interferon-γ (IFN-γ) were measured using a multiplex detection kit (Bio-Rad, Millipore, Hercules, CA, USA). Hepatic lipid levels were determined using the same enzymatic kit used in the plasma analyses after extraction using the method described by Folch et al. [16].

### 2.4. Morphology of Liver, Fat, and Pancreatic Tissues

The liver, epididymal adipose, and pancreatic tissues were harvested from each mouse. Liver and epididymal adipose tissue samples were subsequently fixed in 10% (*v*/*v*) paraformaldehyde/phosphate buffered saline (PBS) and embedded in paraffin for staining with hematoxylin and eosin, and Masson’s trichrome stain. The pancreatic tissue samples were fixed in 4% (*v*/*v*) paraformaldehyde/PBS and embedded in paraffin for immunohistochemical staining of insulin and glucagon. The stained area was visualized using a microscope with 200× magnification.

### 2.5. Hepatic Enzyme Activities and Glycogen Concentrations

Hepatic mitochondrial, cytosolic, and microsomal fractions were prepared as previously described [17] and the protein concentrations were determined via Bradford assay. Fatty acid synthase (FAS) [18], phosphatidate phosphohydrolase (PAP) [19], carnitine palmitoyltransferase (CPT) [20], and β-oxidation [21] activities were measured as previously described. Microsomal 3-hydroxy-3-methyl-glutaryl-coenzyme A reductase (HMGCR) [22] and acyl-CoA:cholesterol acyltransferase (ACAT) [23] activities were measured using [14C]-HMG-CoA and [14C]-oleoyl CoA substrates, respectively. Spectrophotometric assays were used to determine glucose-6-phosphatase (G6Pase) [24], glucokinase [25], and phosphoenolpyruvate carboxykinase (PEPCK) [26] activities as described previously. Hepatic glycogen [27] concentrations were also determined as previously described.

### 2.6. Hepatic H_2_O_2_ and Erythrocyte Thiobarbituric Acid Reactive Substance (TBARS) Concentrations, and Plasma Paraoxonase (PON) Activity

The hepatic H_2_O_2_ [28] and erythrocyte TBARS [29] levels were determined as previously described. The plasma PON activity was measured using the method described by Mackness [30].

### 2.7. RNA Preparation and Quality Control

Total RNA was extracted from adipose tissue, liver, and skeletal muscle using TRIzol reagent (Invitrogen Life Technologies, Grand Island, NY, USA) according to the manufacturer’s instructions. DNase digestion and subsequent re-precipitation in ethanol were performed to remove any DNA and phenol contaminants, respectively. For quality control, the RNA purity and integrity were evaluated using an Agilent 2100 Bioanalyzer (Agilent Technologies, Santa Clara, CA, USA). For the liver, epididymal adipose tissue, and skeletal muscle samples, three pooled RNA sample sets were constructed for each of the ND, HFD, and UR groups as previously described [31]. RNA was stored at −70 °C prior to further analysis via microarray and quantitative polymerase chain reaction (qPCR).

### 2.8. Quantitative Real-Time PCR

Total RNA (1 μg) was reverse-transcribed into cDNA using the QuantiTect^®^ reverse transcription kit (Qiagen, Hilden, Germany), and mRNA expression was quantified via qPCR using the SYBR green PCR kit (Qiagen) and the CFX96TM real-time system (Bio-Rad). Gene-specific mouse primers were used as mentioned in Table S1. Ct values were normalized to those of glyceraldehyde 3-phosphate dehydrogenase (GAPDH) and the relative gene expression was calculated using the 2^−ΔΔ^ Ct method [32].

### 2.9. Microarray Analysis

Total RNA was amplified and purified using the Ambion Illumina RNA amplification kit (Ambion, Waltham, MA, USA). Biotinylated cRNA (750 ng per sample) was hybridized to Illumina MouseWG-6 v2 Expression BeadChips (Illumina, San Diego, CA, USA) according to the manufacturer’s instructions. Hybridized arrays were washed and stained with Amersham Fluorolink streptavidin-Cy3 (GE Healthcare Bio-Sciences, Little Chalfont, UK) following the standard protocol provided in the bead array manual. Hybridization quality and overall chip performance were determined by visual inspection of both the internal quality controls and the raw scanned data in the Illumina BeadStudio software. Probe signal intensities were quantile-normalized and log-transformed. Microarray analysis was performed using the ArrayAssists software (Stratagene, La Jolla, CA, USA) in the R programming language.

The statistical differential gene expression analysis between groups was performed using the non-parametric RankProd approach. Oligonucleotides that presented changes between groups with a false discovery rate (FDR) < 0.05 were considered significant. The Kyoto Encyclopedia of Genes and Genomes (KEGG) pathways mapper (www.genome.jp/kegg) was consulted for the analysis of gene functions involved in circadian rhythm, lipid metabolism, tricarboxylic acid (TCA) cycle, oxidative phosphorylation, and ECM–receptor interaction. These microarray data were deposited in the Gene Expression Omnibus (GEO) database (GEO accession numbers: GSE120243).

### 2.10. Statistical Analysis

The parameter values are expressed as means (standard error of the mean, SEM). Significant differences between the groups were determined via Student’s *t*-test and Wilcoxon *t*-test using the SPSS software (SPSS Inc., Chicago, IL, USA). Results were considered statistically significant at *p* < 0.05.

## 3. Results

### 3.1. UR Decreased Adiposity and Plasma Lipids Levels

**Figure 1:**
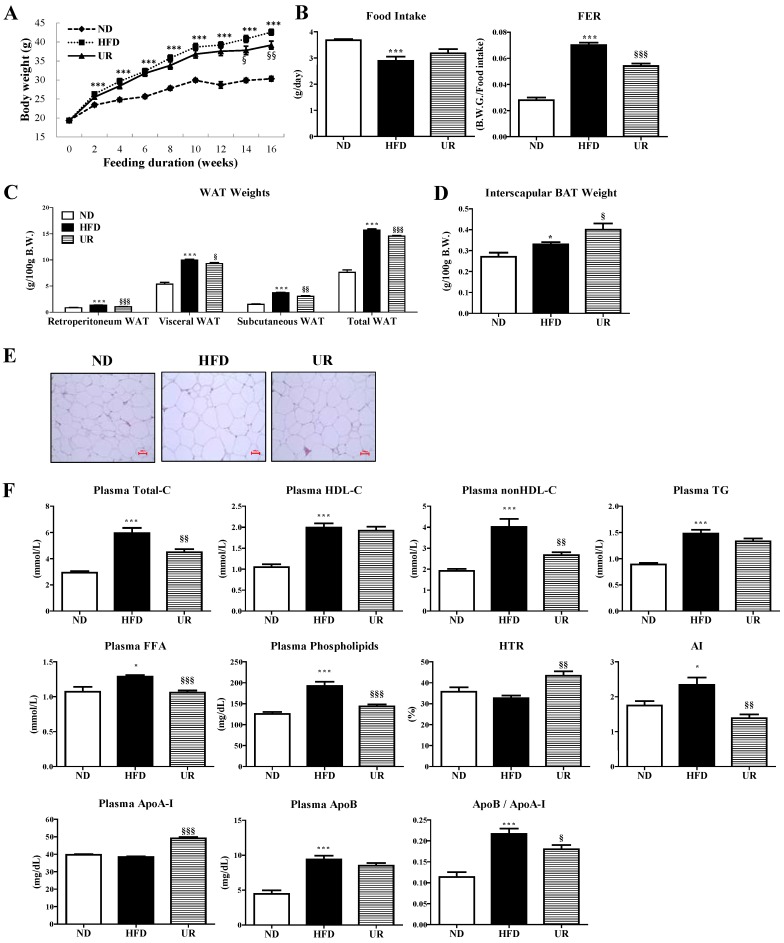
Effect of ursolic acid (UR) treatment on changes: in body weight (BW) (**A**); food intake and food efficiency ratio (**B**); white adipose tissue (WAT) weights (**C**); brown adipose tissue (BAT) weight (**D**); WAT morphology (magnification ×200) (**E**); and plasma lipid levels (**F**) in C57BL/6J mice fed a high-fat diet (HFD; 20% fat, 1% cholesterol). Data are shown as means ± SEM. Normal diet (ND; AIN-76) vs. HFD; * *p* < 0.05, ** *p* <0.01, *** *p* < 0.001. HFD vs. UR (HFD + 0.05% UR); ^§^ *p* < 0.05, ^§§^ *p* < 0.01, ^§§§^ *p* < 0.001. FER, food efficiency ratio, body weight gain/energy intakes per day; WAT, white adipose tissue; BAT, brown adipose tissue; Total-C, total-cholesterol; HDL-C, HDL-cholesterol; TG, triglyceride; FFA, free fatty acid; HTR, ratio of HDL-C to TC; AI, atherogenic index; Apo, apolipoprotein.

UR markedly decreased BW and food efficiency ratio (FER) after Week 14 compared to that of mice in the HFD group (Figure 1A,B). UR also significantly reduced the weights of white adipose tissue (WAT) depots, including retroperitoneal, subcutaneous, visceral (sum of epididymal, perirenal, mesenteric, and retroperitoneal WAT), and total WAT (sum of visceral, subcutaneous, and interscapular WAT), while increasing the interscapular brown adipose tissue (BAT) weight compared with those of mice in the HFD group (Figure 1C,D). Moreover, morphological observations revealed that the epididymal adipocyte size in mice in the UR group was smaller than that in mice in the HFD group (Figure 1E). Plasma levels of TC, non-HDL-C, FFA, phospholipid, AI, and apoB-to-apoAI ratio were significantly lower, whereas the apoA-I level and HTR were significantly higher in mice in the UR group than those in the HFD group (Figure 1F).

### 3.2. UR Abated HFD-Induced Hepatic Steatosis by Modulating Hepatic Lipid-Regulating Enzyme Activities

**Figure 2:**
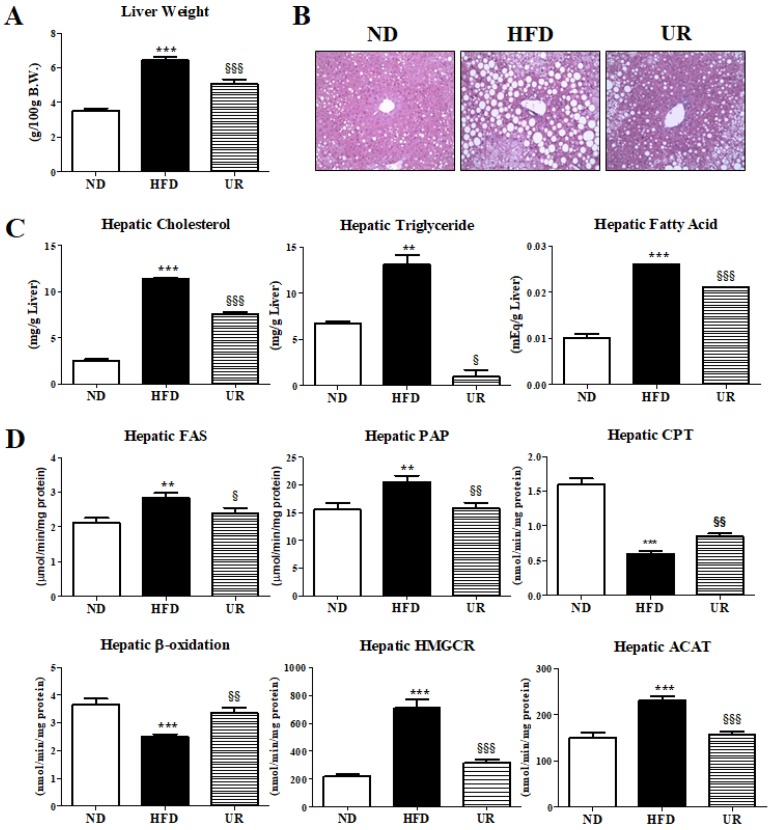
Effect of ursolic acid (UR) treatment on: the liver weight (**A**); hepatic morphology (magnification ×200) (**B**); hepatic lipid levels (**C**); and hepatic lipid-regulating enzyme activities (**D**) in C57BL/6J mice fed a high-fat diet (HFD). Data are shown as means ± SEM. Normal diet (ND; AIN-76) vs. HFD; * *p* < 0.05, ** *p* < 0.01, *** *p* < 0.001. HFD vs. UR (HFD + 0.05% UR); ^§^ *p* < 0.05, ^§§^ *p* < 0.01, ^§§§^ *p* < 0.001. FAS, fatty acid synthase; PAP, phosphatidate phosphohydrolase; CPT, carnitine palmitoyltransferase; HMGCR, 3-hydroxy-3-methyl-glutaryl-coenzyme A reductase; ACAT, acyl-CoA:cholesterol acyltransferase.

UR treatment significantly decreased the liver weight (Figure 2A) as well as the accumulation and size of hepatic lipid droplets relative to that of mice in the HFD group (Figure 2B). Furthermore, treatment with UR markedly decreased the hepatic cholesterol, TG, and fatty acid (FA) contents, relative to that of mice in the HFD group (Figure 2C). The activities of the hepatic enzymes involved in lipogenesis (i.e., FAS and PAP) were significantly reduced, and those related to FA oxidation (i.e., CPT and β-oxidation) were markedly increased by UR treatment compared to those in mice in the HFD group (Figure 2D). Activities of the hepatic cholesterol-regulating enzymes HMGCR and ACAT were also remarkably lower in mice in the UR group than those in mice in the HFD group (Figure 2D).

### 3.3. UR Ameliorated Tissue Fibrosis in Mice with DIO

**Figure 3:**
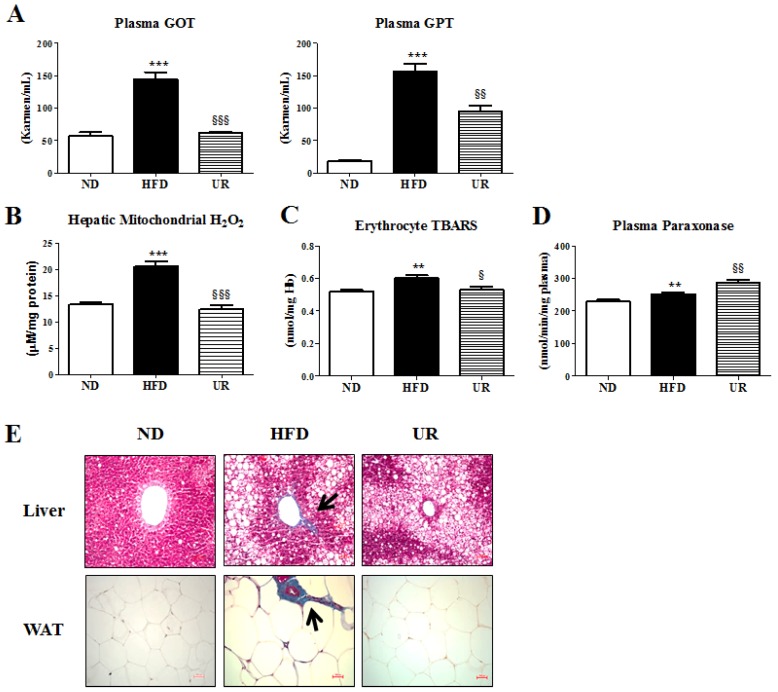
Effect of ursolic acid (UR) treatment on: plasma GOT and GPT (**A**); hepatic mitochondrial H_2_O_2_ (**B**); erythrocyte thiobarbituric acid reactive substance (TBARS) (**C**); plasma paraoxonase (PON) (**D**); and Masson’s trichrome staining (magnification ×200) (**E**) in C57BL/6J mice fed a high-fat diet (HFD). Data are shown as means ± SEM. Normal diet (ND; AIN-76) vs. HFD; * *p* < 0.05, ** *p* < 0.01, *** *p* < 0.001. HFD vs. UR (HFD + 0.05% UR); ^§^ *p* < 0.05, ^§§^ *p* < 0.01, ^§§§^ *p* < 0.001. GOT, glutamic oxaloacetic transaminase; GPT, glutamic pyruvic transaminase; TBARS, thiobarbituric acid reactive substance.

Levels of hepatic lipotoxicity markers including plasma GOT and GPT were significantly decreased by UR treatment compared with those in mice in the HFD group (Figure 3A). Hepatic mitochondrial H_2_O_2_ and erythrocyte TBARS levels were also significantly lower in mice in the UR group than in mice in the HFD group (Figure 3B,C). The activity of plasma PON, an atheroprotective protein, was strikingly increased in mice in the UR group compared to that in mice in the HFD group (Figure 3D). To assess tissue fibrosis, we used Masson’s trichrome stain, which colors fibrous collagen-containing tissue blue, to display the fibrous tissue of the liver and adipose tissue in the HFD group. The hepatic portal vein of HFD-fed mice was surrounded by fibrous bands, and epididymal WAT depots in HFD-fed mice contained pronounced trichrome-positive “streaks” interspersed among the adipocytes. The UR group, on the other hand, showed normal hepatic architecture and fat-pad, with very thin, closely packed collagen sheets surrounding each adipocyte (Figure 3E).

### 3.4. UR Prevented HFD-Induced IR and Pancreatic Islet Hypertrophy

**Figure 4:**
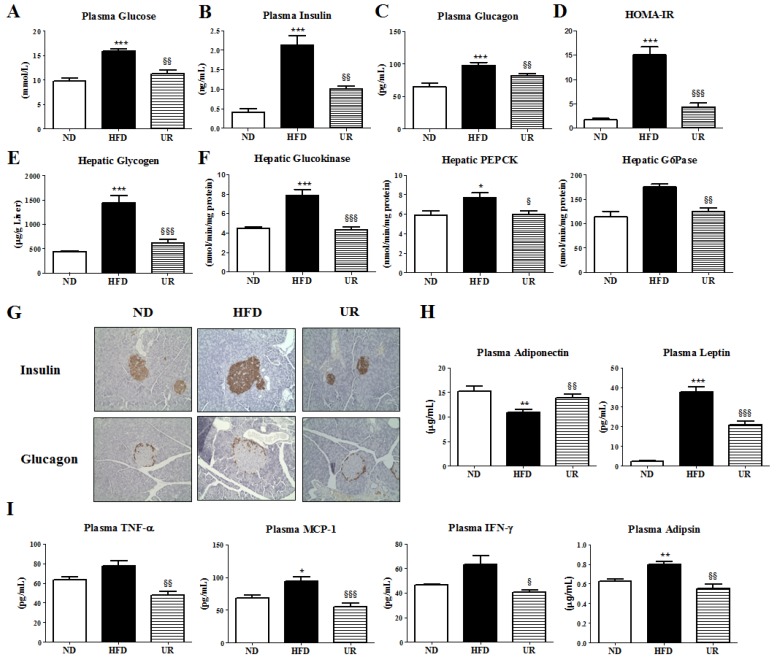
Effect of ursolic acid (UR) treatment on: plasma glucose level (**A**); insulin level (**B**); glucagon level (**C**); homeostasis model assessment-estimated insulin resistance (HOMA-IR) index (**D**); hepatic glycogen content (**E**); hepatic glucose-regulating enzyme activities (**F**); immunohistochemical staining of the pancreatic tissue (**G**); plasma adipokine (**H**); and pro-inflammatory cytokine levels (**I**) in C57BL/6J mice fed a high-fat diet (HFD). Data are shown as means ± SEM. Normal diet (ND; AIN-76) vs. HFD; * *p* < 0.05, ** *p* < 0.01, *** *p* < 0.001. HFD vs. UR (HFD + 0.05% UR); ^§^ *p* < 0.05, ^§§^ *p* < 0.01, ^§§§^ *p* < 0.001. HOMA-IR, homeostasis model assessment for insulin resistance; PEPCK, phosphoenolpyruvate carboxykinase; G6Pase, glucose-6-phosphatase; TNF-α, tumor necrosis factor alpha; MCP-1, monocyte chemoattractant protein-1; IFN-γ, interferon gamma.

UR significantly decreased plasma glucose, insulin, and glucagon levels, and HOMA-IR index relative to that in mice in the HFD group (Figure 4A–D). Glycogen levels and glucokinase, PEPCK, and G6Pase activities were lowered by UR compared with those in the liver of mice in the HFD group (Figure 4E,F). Immunohistochemical staining of the pancreatic tissue for insulin and glucagon also revealed that pancreatic islet hypertrophy caused by HFD was prevented by UR treatment, thereby normalizing the insulin and glucagon content in the plasma (Figure 4G). Moreover, expression of the anti-inflammatory cytokine adiponectin was markedly increased, whereas levels of pro-inflammatory cytokines/chemokines such as leptin, TNF-α, MCP-1, IFN-γ, and adipsin were significantly suppressed by UR treatment relative to that in mice in the HFD group (Figure 4H,I).

### 3.5. UR Altered the Transcriptional Responses Involved in Circadian Clock and ECM in the Liver and Muscle Tissue of DIO Mice

**Figure 5:**
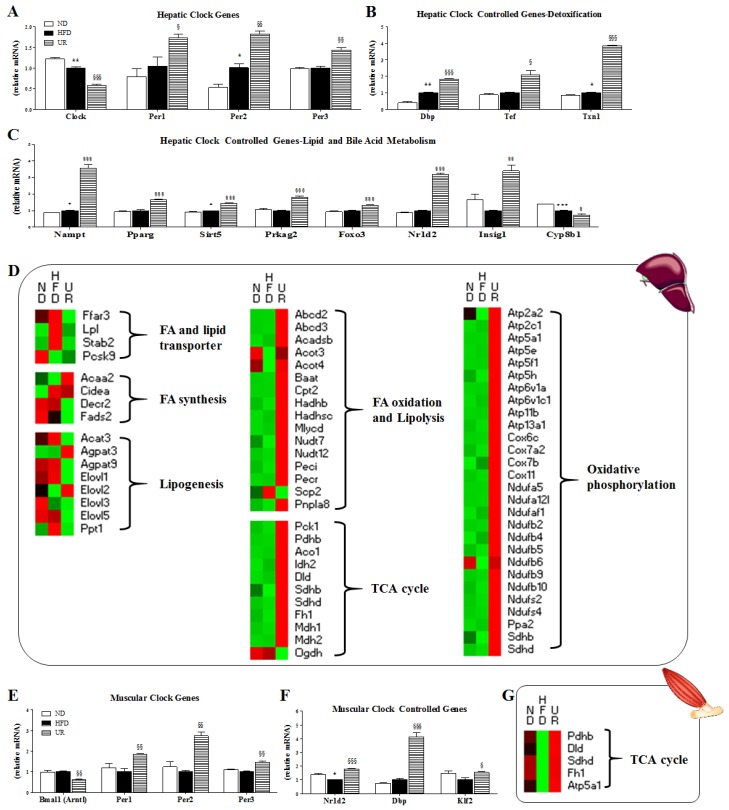
Effect of ursolic acid (UR) treatment on: the expression of hepatic clock genes in detoxification (**A**); clock-controlled genes (CCGs) involved in detoxification (**B**); lipid and bile acid metabolism (**C**); transcription patterns of hepatic genes related to lipid metabolism (**D**); muscular clock genes (**E**); CCGs (**F**); and tricarboxylic acid (TCA) cycle-associated genes (**G**). Symbols in red indicate genes that were up-regulated while those in green denote genes that were down-regulated.

Microarray analysis revealed that 1773 genes in the liver, 106 genes in WAT, and 202 genes in the muscle were differentially expressed in response to UR treatment compared to those in HFD-fed mice (Figures S1 and S2). In the liver, UR altered the expression of clock genes and CCGs. Among them, UR reduced Clock gene expression, while enhancing the expression of *Per1*, *Per2*, and *Per3* genes when compared to that in mice in the HFD group (Figure 5A). The expression of genes responsible for detoxification (*Dbp*, *Tef*, and *Txn1*), as well as lipid and bile acid metabolism (*Nampt*, *Pparg*, *Sirt5*, *Prkag2*, *Foxo3*, *Nr1d2*, and *Insig1*) was increased, while expression of the bile acid biosynthesis-related gene *Cyp8b1* was down-regulated by UR (Figure 5B,C). With regard to lipid metabolism, UR lowered the expression of hepatic genes up-regulated by HFD and involved in FA and lipid transport, FA synthesis, and lipogenesis, and enhanced the expression of genes associated with FA oxidation and lipolysis, the TCA cycle, and oxidative phosphorylation (Figure 5D and Table S2). Microarray analysis of the muscle also revealed that UR altered the expression of clock and CCGs, and their transcription regulators. UR treatment decreased Arntl (Bmal1) gene expression and increased that of PER family genes (*Per1*, *Per2*, and *Per3*), CCGs (*Nr1d2*, *Dbp*, and *Klf2*), and TCA cycle-related genes (*Pdhb*, *Dld*, *Sdhb*, *Fh1*, and *Atp5a1*), consistent with our hepatic microarray analysis results (Figure 5E–G and Table S3).

**Figure 6:**
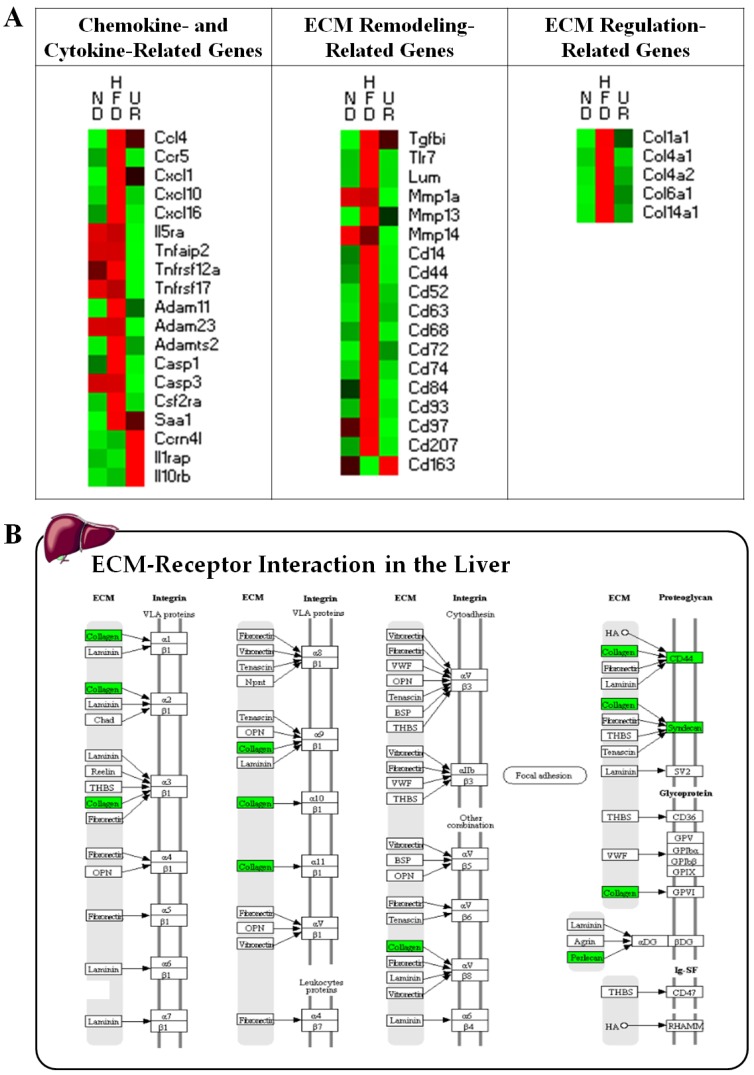
Effect of ursolic acid (UR) treatment on the transcription patterns of hepatic genes related to chemokine/cytokine expression, extracellular matrix (ECM) remodeling, and ECM regulation (**A**); and ECM–receptor interaction (**B**). Symbols in red indicate genes that were up-regulated while those in green denote genes that were down-regulated. UR down-regulated collagen, Cd44, Syndecan and Perlecan gene expressions involved in ECM-receptor interaction compared to HFD.

Next, we examined the levels of many key fibrotic genes involved in cytokine/chemokine expression, ECM remodeling, and ECM regulation during HFD-induced inflammation (Figure 6 and Tables S4 and S5). With regard to inflammatory pathways involved in obesity, UR down-regulated the hepatic transcription of several pro-inflammatory chemokines/cytokines (*Ccl4*, *Ccr5*, *Cxcl1*, *Cxcl10*, *Cxcl16*, *Il5ra*, *Tnfaip2*, *Infrsf12a*, *Tnfrsf17*, *Adam11*, *Adam23*, *Adamts2*, *Casp1*, *Casp3*, *Csf2ra*, and *Saa1*), while up-regulating the expression of *Ccrn4l*, *Il1rap*, and *Il10rb* genes. The expression of most of the genes responsible for ECM remodeling and regulation was up-regulated by consumption of the HFD, whereas these genes were down-regulated by UR treatment. Specifically, ECM remodeling-associated genes (*Tgfbi*, *Tlr7*, *Lum*, *Mmp1a*, *Mmp13*, *Cd14*, *Cd44*, *Cd52*, *Cd63*, *Cd68*, *Cd72*, *Cd74*, *Cd84*, *Cd93*, and *Cd207*) and ECM regulation-associated genes (*Col1a1*, *Col4a1*, *Col4a2*, *Col6a1*, and *Col14a1*) were down-regulated, while expression of the M2 macrophage marker *Cd163* was markedly up-regulated by UR in comparison to that in mice in the HFD group (Figure 6A and Table S4). Moreover, pathway analysis using the KEGG mapper also revealed that the expression of genes involved in ECM–receptor interactions was down-regulated by UR (Figure 6B).

In contrast to our hepatic microarray results, UR increased the expression of muscular genes involved in cytokine/chemokine expression (*Adam9*, *Cxcl4*, *Il15*, *Itgbl1*, and *Itgb1bp3*), ECM remodeling (*Dcn*, *Sparc*, and *Sparcl1*), and ECM regulation (*Col1a1*, *Col3a1*, *Col5a1*, *Col6a1*, *Col6a3*, *Col8a1*, and *Col16a1*; Table S5).

## 4. Discussion

In the current study, we evaluated the effects of UR and the molecular mechanisms underlying its metabolic regulatory properties in DIO and metabolic disease via transcriptomic analysis. This study demonstrates for the first time that dietary UR improved inflammation-mediated metabolic diseases including DIO, hepatic steatosis, dyslipidemia, fibrosis, and IR by controlling the circadian clock and suppressing hepatic ECM remodeling in DIO mice. The following observations were made: (a) UR improved hepatic steatosis by restricting TG availability and decreasing lipogenesis, while promoting lipolysis, TCA cycle, and oxidative phosphorylation in the liver; (b) oligonucleotide microarray profiling revealed that hepatic transcriptomic changes in response to UR treatment were more dynamic than those in WAT and muscle, and that UR altered the expression of various clock and CCGs, which may be linked to the improvement of hepatic steatosis and fibrosis by controlling lipid metabolism and enhancing detoxification; (c) UR reduced excessive reactive oxygen species (ROS) production via an increase in plasma PON activity, adipokine/cytokine dysregulation, and ECM accumulation in the liver, which also contributed to improvement of hepatic lipotoxicity and fibrosis; and (d) UR treatment improved and normalized pancreatic islet dysfunction, and suppressed hepatic gluconeogenesis, thereby reducing obesity-associated IR in HFD-fed mice.

UR exerts various biological effects, including anti-inflammatory, anti-oxidative, anti-cancer, and anti-obesity activities [10,11,12,13,14,15]. In rat models of DIO, UR administration has been reported to ameliorate HFD-induced hepatic steatosis and inflammatory hyperalgesia by activating the PPAR-α pathway [10,11], and prevent obesity and IR by increasing FFA burning through enhancing skeletal muscle FFA uptake and β-oxidation via an uncoupling protein 3 (UCP3)/AMPK-dependent pathway [12]. In HFD-fed Swiss mice, administration of aqueous UR (50 mg/L) for 15 weeks decreased BW, visceral adiposity, and blood glucose and plasma lipid levels [13]. In addition, treatment with UR (0.27% *w*/*w*) for three weeks increased energy expenditure in skeletal muscle and brown fat, thus preventing obesity, glucose tolerance, and hepatic steatosis in C57BL/6J HFD-fed mice [14]. However, to date, few studies have been conducted on the effects of long-term UR treatment based on the integration of transcriptional profiles and phenotypic biomarkers. In the current study, we performed microarray analysis on three murine tissues (liver, muscle, and epididymal WAT) to examine the effects and potential underlying mechanisms of long-term dietary UR supplementation on HFD-induced obesity and associated metabolic diseases, including hepatic steatosis, fibrosis, IR, dyslipidemia, and inflammation in C57BL/6J mice. Oligonucleotide microarray profiling revealed that, among the three organs, the liver transcriptome was the most significantly altered by UR, revealing changes approximately 8.8 and 16.7 times higher than those in muscle and WAT, respectively, suggesting that UR is a more effective regulator of the liver transcriptome.

In this study, our global transcriptomic analyses showed that canonical pathways associated with the “circadian rhythm” and “ECM–receptor interaction” were effectively regulated by UR in the liver. The circadian clock is an endogenous biological timekeeping system that synchronizes physiology and behavior to day/night cycles. Interactions between core clock transcription factors (CLOCK and BMAL1) and repressor Period (*Per*) and Cryptochrome (*Cry*) genes, as well as a spectrum of CCGs, are known to play essential roles in the metabolism of glucose, bile acids, lipids, and cholesterol in the liver [5]. Disruption of genetic or environmental factors such as energy imbalance and the circadian clock can cause metabolic diseases including hepatic steatosis, fibrosis, and diabetes, or exacerbate pathological states [5,6,7,8,9]. In this study, UR increased the expression of Per (*Per1*, *Per2*, and *Per3*) mRNA, and decreased that of clock genes, thereby modulating the expression of various CCGs involved in detoxification (*Dbp*, *Tef*, and *Txn1*), and lipid and bile acid metabolism (*Nampt*, *Pparg*, *Sirt5*, *Prkag2*, *Foxo3*, *Nr1d2*, and *Insig1*). Circadian clocks mediate protective responses to oxidative stress [33], and albumin D-site-binding protein (DBP), thyrotroph embryonic factor (TEF), and thioredoxin (TXN), the master regulators of hepatic detoxification enzymes, are rhythmically controlled by clock genes [34]. Consistent with the increased expression of detoxification genes observed in UR-supplemented mice, we observed decreased excessive ROS production as evidenced by reduced hepatic H_2_O_2_ and erythrocyte TBARS levels, as well as enhanced plasma PON activity. It is well known that ROS production contributes to fibrosis and IR, and various studies support the concept that molecular clock output genes involved in detoxification, such as *Dbp* and *Txn*, perform essential functions in the development of metabolic diseases [7,35]. In addition, UR decreased the expression of genes associated with chemokine/cytokine expression, ECM remodeling, and ECM regulation that were up-regulated by HFD, and which are known to contribute to fibrosis. Thus, these observations indicate that UR has the potential to regulate hepatic clock genes and CCGs involved in detoxification, which is linked to reduced pro-inflammatory responses, ECM accumulation in the liver, and increased insulin sensitivity, thereby preventing hepatic lipotoxicity, fibrosis, and IR in DIO mice.

CCGs altered by UR treatment were also strongly linked to hepatic circadian lipid metabolism. Among CCGs, UR increased the mRNA expression of *Sirt5*, *Prkag2* (AMPK), and *Foxo3* involved in lipolysis and FA oxidation, and decreased *Cyp8b1* mRNA expression involved in bile acid synthesis compared to that in mice in the HFD group, which in turn induced a significant increase in the hepatic TCA cycle and oxidative phosphorylation, which may contribute to the reduction of hepatic steatosis. Other core clock genes, such as *Nampt*, *Pparg*, *Nr1d2*, and *Insig1*, were also significantly up-regulated by UR. Previous studies have reported that *Nampt* mRNA is decreased in the liver of human subjects with non-alcoholic fatty liver disease, and *Nampt* has been shown to protect hepatocytes by increasing SIRT1 activity [36,37]. *Nr1d2* also represses the expression of hepatic *Apoc3* mRNA, a risk factor for atherosclerosis [38], and hepatic *Insig1* overexpression reduces lipogenesis via decreased expression of *Srebp1c* mRNA and its target enzymes [39]. Interestingly, UR increased *Pparg* and *Nampt* mRNA expression. Stromsdorfer et al. reported that the loss of nicotinamide phosphoribosyltransferase (NAMPT) impairs adipose tissue function and decreases PPARγ expression by increasing its phosphorylation, and in turn decreases adiponectin production, while increasing FFA production, which causes IR in adipose tissue, liver, and skeletal muscle [40]. Therefore, it is plausible that a UR-mediated increase in hepatic *Nampt* mRNA expression could increase *Pparg* expression, which in turn reduces plasma FFA and increases adiponectin, thereby ameliorating hepatic steatosis and IR.

Consistent with the hepatic microarray results, UR increased Per (*Per1*, *Per2*, and *Per3*) mRNA expression, and diminished *Arntl* (BMAL1) gene expression in the skeletal muscle, thereby increasing muscular expression of CCGs including *Nr1d2*, *Dbp*, and *Klf2*, and TCA cycle-associated genes. However, in contrast to that in the liver, mRNA expression involved in chemokine/cytokine expression, ECM remodeling, and ECM regulation was increased rather than decreased by UR supplementation. Our findings contrast somewhat with previous studies demonstrating greater ECM accumulation in skeletal muscle in obese insulin-resistant models [41,42]. Our observations after UR treatment may be linked with ECM homeostasis in the muscle, although this would require additional experiments for verification. Taken together, the present findings suggest that UR treatment attenuates hepatic steatosis, fibrosis, and IR by modulating liver-specific metabolic responses, in connection with circadian regulation.

## 5. Conclusions

In summary, the data obtained from our animal study indicate that UR can suppress DIO and modulate obesity-associated metabolic disorders including hepatic steatosis, fibrosis, and IR by modulating liver-specific metabolic responses such as “ECM–receptor interactions” under circadian control. Moreover, considering that no effective treatment for liver fibrosis is currently available, therapeutic approaches targeting hepatic circadian clock genes and CCGs using UR may ameliorate the deleterious effects of DIO and complications associated therewith, such as hepatic fibrosis. However, these observations require additional experiments for verification, because translation of results obtained in an animal model to a human population is not completely reflected.

## Supplementary Materials

The following are available online at http://www.mdpi.com/2072-6643/10/11/1719/s1, Figure S1: The number of differentially expressed genes (DEGs) in the liver, adipose tissue and skeletal muscle of C57BL/6J mice. Differentially expressed genes based on comparison of HFD vs. ND and UR vs. HFD according to *p*-value < 0.05, log2 fold change > 0.5, Figure S2: Real-time RT-qPCR validation. Microarray and RT-qPCR data shown as means ± S.E. ND vs. HFD; * *p* < 0.05, ** *p* < 0.01, *** *p* < 0.001. HFD vs. UR; ^§^ *p* < 0.05, ^§§^ *p* < 0.01, ^§§§^ *p* < 0.001. White bar = ND group; black bar = HFD group; stripe bar = UR group, Table S1: Primer sequences used for RT-qPCR validation of the microarray data, Table S2: Effect of ursolic acid on transcriptional pattern of lipid metabolism-related genes in liver of C57BL/6J mice, Table S3: Effect of ursolic acid on transcriptional pattern of TCA cycle-related genes in muscle of C57BL/6J mice, Table S4: Effect of ursolic acid on transcriptional pattern of fibrosis-related genes in liver of C57BL/6J mice. Table S5: Effect of ursolic acid on transcriptional pattern of fibrosis-related genes in muscle of C57BL/6J mice.
