# Supplementary material for: Ursolic Acid Attenuates Hepatic Steatosis, Fibrosis, and Insulin Resistance by Modulating the Circadian Rhythm Pathway in Diet-Induced Obese Mice
Source: Nutrients. 2018 Nov 9;10(11):1719. doi: 10.3390/nu10111719 (PMC6266464; doi:10.3390/nu10111719)
Supplement: Supplementary file 1 [file nutrients-10-01719-s001.pdf]

Ursolic acid attenuates hepatic steatosis, fibrosis, and insulin resistance by modulating the circadian rhythm pathway in diet-induced obese mice

Su-Kyung Shin [1,†], Eun-Young Kwon [2,3,†] and Myung-Sook Choi [2,3,*]

1. Materials and Methods

Table S1. Primer sequences used for RT-qPCR validation of the microarray data.

Gene	Primer direction	Primer sequence
Glyceraldehyde 3-phosphate dehydrogenase (Gapdh)	Forward	5′-AGGTCGGTGTGAACGGATTTG-3′
	Reverse	5′-TGTAGACCATGTAGTTGAGGTCA-3′
Complement factor D (adipsin) (Cfd)	Forward	5′-GCCGACCTGACAGCCTTGAG-3′
	Reverse	5′-TTCCACTTCTTTGTCCTCGTATTGC-3′
3-hydroxy-3-methylglutaryl coenzyme A reductase (Hmgcr)	Forward	5′-TTCACGCTCATAGTCGCTGGATAG-3′
	Reverse	5′-TGGTTCAATTCTCTTGGACACATCTTC-3′
Acetyl Coenzyme A Acetyltransferase 1 (Acat1)	Forward	5′-CTCACGGCAGGAACAGGATACG-3′
	Reverse	5′-TTCTTCATCTTCTTTCACCACCACATC-3′
Cell death-inducing DFFA-like effector A (Cidea)	Forward	5′-TTTCAAACCATGACCGAAGTAGC-3′
	Reverse	5′-CCTCCAGCACCAGCGTAACC-3′
Lipoprotein lipase (Lpl)	Forward	5′-CAAGTTCAACGGCACAGTCAAGG-3′
	Reverse	5′-ACATACTCAGCACCAGCATCACC-3′

2. Results

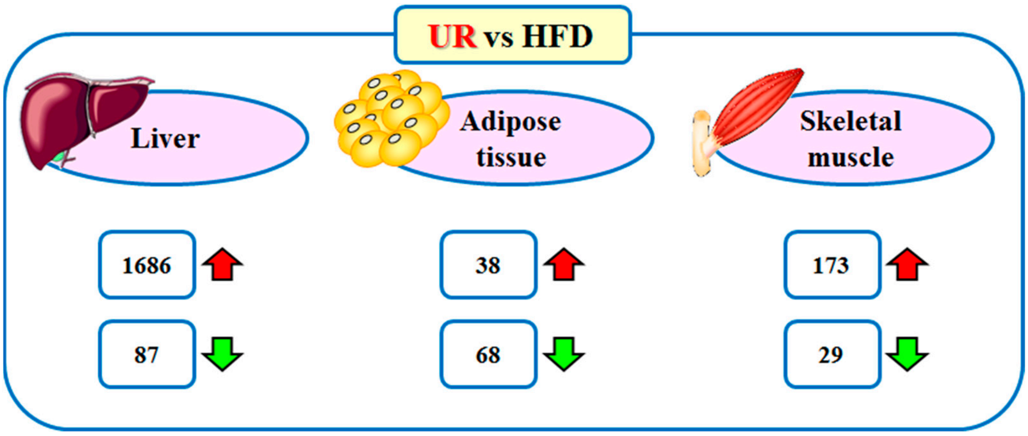

Figure S1. The number of differentially expressed genes (DEGs) in the liver, adipose tissue and skeletal muscle of C57BL/6J mice. Differentially expressed genes based on comparison of HFD vs. ND and UR vs. HFD according to p-value < 0.05, $\log_2$ fold change > 0.5.

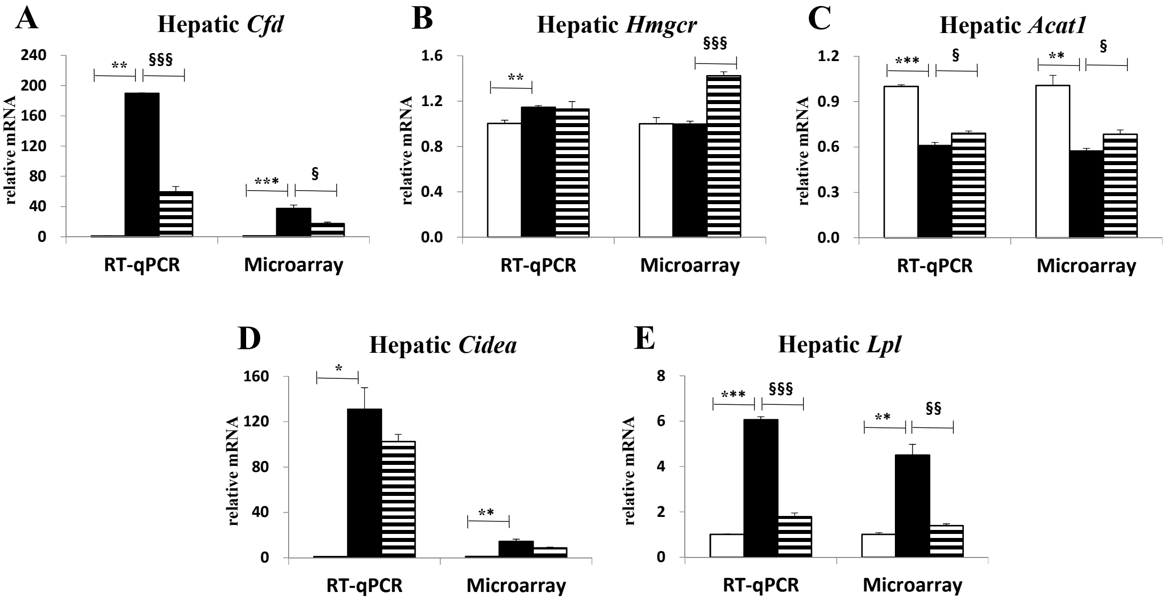

Figure S2. Real-time RT-qPCR validation. Microarray and RT-qPCR data shown as means ± S.E. ND vs HFD; *p<0.05, **p<0.01, ***p<0.001. HFD vs UR; §p<0.05, §§p<0.01, §§§p<0.001. White bar = ND group; black bar = HFD group; stripe bar = UR group.

Table S2. Effect of ursolic acid on transcriptional pattern of lipid metabolism-related genes in liver of C57BL/6J mice.

Gene	Fold change (HFD/ND)	*p* value	Fold change (UR/HFD)	*p* value	Heatmap
FA and lipid transporter					
Ffar3	1.25	0.01565	-1.68	0.00000	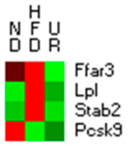
Lpl	4.44	0.00000	-3.20	0.00000	
Stab2	1.39	0.00003	-1.44	0.00001	
Pcsk9	-2.97	0.00047	1.41	0.00000	
FA synthesis					
Acaa2	-1.26	0.01237	1.73	0.00000	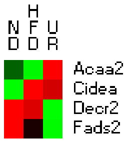
Cidea	14.08	0.00000	-1.67	0.04045	
Decr2	-1.10	0.92599	-1.63	0.00001	
Fads2	-1.54	0.00011	-1.56	0.00007	
Lipogenesis					
Acat3	1.17	0.29511	-1.42	0.00023	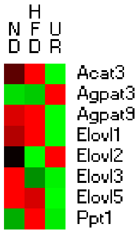
Agpat3	1.09	0.95761	2.85	0.00000	
Agpat9	1.05	0.99653	-1.37	0.00029	
Elovl1	1.09	0.68989	-1.36	0.00005	
Elovl2	-1.93	0.00005	3.69	0.00000	
Elovl3	-3.02	0.00002	-1.67	0.01960	
Elovl5	-1.09	0.93562	-1.47	0.00006	
Ppt1	1.32	0.00066	-1.43	0.00002	
FA oxidation and Lipolysis					
Abcd2	-1.00	1.00000	1.67	0.00000	
Abcd3	-1.05	1.00000	3.96	0.00000	
Acadsb	1.17	0.67896	4.01	0.00000	
Acot3	-2.96	0.00007	2.15	0.00542	

Acot4	-1.66	0.00050	2.02	0.00000
Baat	-1.04	0.99999	4.06	0.00000
Cpt2	1.00	1.00000	1.75	0.00000
Hadhb	-1.18	0.89958	3.66	0.00000
Hadhsc	1.04	0.99880	1.50	0.00000
Mlycd	1.03	0.99933	1.83	0.00000
Nudt7	-1.30	0.70138	2.40	0.00010
Nudt12	-1.04	0.99929	1.78	0.00000
Peci	-1.13	0.80726	2.57	0.00000
Pecr	1.03	1.00000	2.89	0.00000
Scp2	1.26	0.01423	-1.47	0.00003
Pnpla8	-1.06	0.97725	1.47	0.00000
TCA cycle				
Pck1	-1.05	0.99994	1.64	0.00043
Phdb	-1.02	1.00000	2.63	0.00000
Aco1	1.04	0.99993	2.72	0.00000
Idh2	1.28	0.49912	4.34	0.00000
Dld	1.09	0.99913	2.95	0.00000
Sdhb	-1.13	0.22801	1.41	0.00001
Sdhd	-1.12	0.92022	3.73	0.00000
Fh1	-1.17	0.45301	2.92	0.00000
Mdh1	-1.06	0.99835	2.35	0.00000
Mdh2	1.02	1.00000	2.19	0.00000
Ogdh	-1.09	0.85601	-1.41	0.00004
Oxidative phosphorylation				
Apt2a2	-1.33	0.04153	1.74	0.00002
Apt2c1	1.03	0.99917	1.65	0.00000
Atp5a1	-1.01	1.00000	4.75	0.00000
Apt5e	1.03	0.99998	2.00	0.00000
Atp5f1	1.01	1.00000	1.75	0.00003
Atp5h	-1.14	0.17032	1.9	0.00000
Atp6v1a	1.01	1.00000	2.51	0.00000
Atp6v1c1	1.05	0.99972	1.65	0.00001
Atp11b	1.00	1.00000	1.74	0.00000
Atp13a1	-1.06	0.99694	1.91	0.00000
Cox6c	-1.02	1.00000	2.80	0.00000
Cox7a2	-1.18	0.50482	3.76	0.00000
Cox7b	1.16	0.53381	1.48	0.00036
Cox11	-1.04	0.99988	1.92	0.00000
Ndufa5	-1.00	1.00000	2.48	0.00000
Ndufa12l	-1.01	1.00000	2.81	0.00000
Ndufaf1	1.09	0.72080	1.66	0.00000
Ndufb2	-1.02	1.00000	2.67	0.00000
Ndufb4	1.12	0.87708	1.62	0.00004
Ndufb5	-1.14	0.54419	3.02	0.00000
Ndufb6	-1.53	0.00340	1.50	0.00554
Ndufb9	-1.07	0.99825	3.86	0.00000

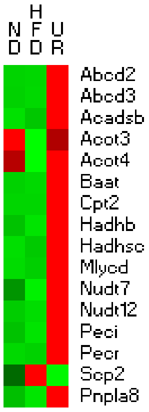

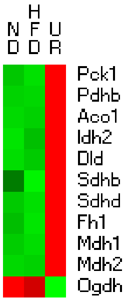

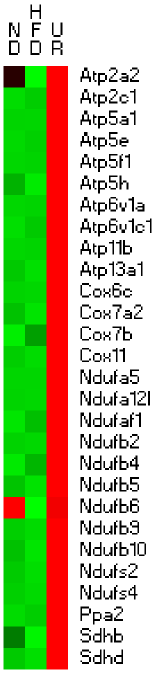

Ndufb10	-1.14	0.05687	2.57	0.00000
Ndufs2	-1.04	0.99994	1.73	0.00001
Ndufs4	-1.06	0.99981	3.83	0.00000
Ppa2	1.06	0.99742	2.88	0.00000
Sdhb	-1.13	0.22801	1.41	0.00001
Sdhd	-1.12	0.92022	3.73	0.00000

Ffar3, free fatty acid receptor 3; Lpl, lipoprotein lipase; Stab2, stabilin 2; Pcsk9, proprotein convertase subtilisin/kexin type 9; Acaa2, acetyl-Coenzyme A acyltransferase 2 (mitochondrial 3-oxoacyl-Coenzyme A thiolase); Cidea, cell death-inducing DNA fragmentation factor, alpha subunit-like effector A; Decr2, peroxisomal 2,4-dienoyl-CoA reductase; Fads2, fatty acid desaturase 2; Acat3, acetyl-Coenzyme A acetyltransferase 3; Agpat, 1-acylglycerol-3-phosphate O-acyltransferase; Elovl, elongation of very long chain fatty acids (FEN1/Elo2, SUR4/Elo3, yeast)-like; Ppt1, palmitoyl-protein thioesterase 1; Abcd, ATP-binding cassette, sub-family D (ALD), member; Acadsb, acyl-Coenzyme A dehydrogenase, short/branched chain; Acot, acyl-CoA thioesterase; Baat, bile acid-Coenzyme A: amino acid N-acyltransferase; Cpt2, carnitine palmitoyltransferase 2; Hadhb, hydroxyacyl-Coenzyme A dehydrogenase/3-ketoacyl-Coenzyme A thiolase/enoyl-Coenzyme A hydratase (trifunctional protein), beta subunit; Mlycd, malonyl-CoA decarboxylase; Nudt, nudix (nucleoside diphosphate linked moiety X)-type motif; Peci, peroxisomal delta3, delta2-enoyl-Coenzyme A isomerase; Pcer, peroxisomal trans-2-enoyl-CoA reductase; Scp2, sterol carrier protein 2; Pnpla8, patatin-like phospholipase domain containing 8; Pck1, phosphoenolpyruvate carboxykinase 1, cytosolic; Phdb, pyruvate dehydrogenase (lipoamide) beta; Aco1, aconitase 1; Idh2, isocitrate dehydrogenase 2 (NADP+), mitochondrial; Dld, dihydrolipoamide dehydrogenase; Fh1, fumarate hydratase 1; Mdh, malate dehydrogenase; Ogdh, oxoglutarate dehydrogenase (lipoamide); Atp2a2, ATPase, Ca++ transporting, cardiac muscle, slow twitch 2; Atp2c1, ATPase, Ca++-sequestering; Apt5a1, ATP synthase, H+ transporting, mitochondrial F1 complex, alpha subunit, isoform 1; Atp5e, ATP synthase, H+ transporting, mitochondrial F1 complex, epsilon subunit ; Atp5f1, ATP synthase, H+ transporting, mitochondrial F0 complex, subunit b, isoform 1; Atp5h, ATP synthase, H+ transporting, mitochondrial F0 complex, subunit d; Atp6v1a, ATPase, H+ transporting, lysosomal V1 subunit A; Atp6v1c1, ATPase, H+ transporting, lysosomal V1 subunit C1; Atp11b , ATPase, class VI, type 11B; Atp13a1, ATPase type 13A1; Cox, cytochrome c oxidase, subunit Vic; Ndufa, NADH dehydrogenase (ubiquinone) 1 alpha; Ndufaf1, NADH dehydrogenase (ubiquinone) 1 alpha subcomplex, assembly factor 1; Ndufb, NADH dehydrogenase (ubiquinone) 1 beta; Ndufs, NADH dehydrogenase (ubiquinone) Fe-S protein; Ppa2, pyrophosphatase (inorganic) 2; Sdhb, succinate dehydrogenase complex, subunit B, iron sulfur (Ip); Sdhd, succinate dehydrogenase complex, subunit D, integral membrane protein.

Table S3. Effect of ursolic acid on transcriptional pattern of TCA cycle-related genes in muscle of C57BL/6J mice.

Gene	Fold change (HFD/ND)	*p* value	Fold change (UR/HFD)	*p* value	Heatmap
TCA cycle					
Pdhb	-1.34	0.02208	1.65	0.00004	
Dld	-1.32	0.01220	1.72	0.00000	
Sdhb	-1.46	0.01201	1.66	0.00045	
Fh1	-1.45	0.00180	1.64	0.00004	
Atp5a1	-1.25	0.18099	1.52	0.00061	

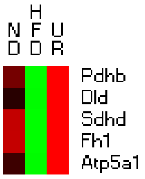

Phdb, pyruvate dehydrogenase (lipoamide) beta; Dld, dihydrolipoamide dehydrogenase; Sdhb, succinate dehydrogenase complex, subunit B, iron sulfur (Ip); Fh1, fumarate hydratase 1; Apt5a1, ATP synthase, H+ transporting, mitochondrial F1 complex, alpha subunit, isoform 1.

Table S4. Effect of ursolic acid on transcriptional pattern of fibrosis-related genes in liver of C57BL/6J mice.

Gene	Fold change (HFD/ND)	*p* value	Fold change (UR/HFD)	*p* value	Heatmap
Chemokines and Cytokines					
Ccl4	2.32	0.00002	-1.45	0.01239	
Ccr5	1.44	0.00132	-1.61	0.00004	
Cxcl1	4.49	0.00000	-2.06	0.00580	
Cxcl10	1.48	0.03471	-1.46	0.04346	
Cxcl16	1.47	0.00481	-1.70	0.00008	
Il5ra	-1.05	0.99939	-1.51	0.00004	
Tnfaip2	-1.00	1.00000	-1.64	0.00001	
Tnfrsf12a	1.21	0.52211	-1.65	0.00043	
Tnfrsf17	-1.09	0.88085	-1.51	0.00001	
Adam11	2.87	0.00000	-1.88	0.00000	
Adam23	-1.01	1.00000	-1.61	0.00004	
Adamts2	1.77	0.00018	-1.52	0.00674	
Casp1	1.35	0.00126	-1.61	0.00000	
Casp3	1.01	1.00000	-1.42	0.00000	
Csf2ra	1.38	0.01484	-1.42	0.00695	
Saa1	5.09	0.00000	-1.97	0.00512	
Ccrn4l	1.27	0.77589	3.80	0.00000	
Il1rap	-1.18	0.73727	3.43	0.00000	
Il10rb	1.08	0.98360	1.52	0.00006	

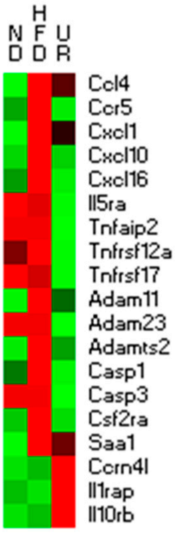

Gene	Fold change (HFD/ND)	*p* value	Fold change (UR/HFD)	*p* value	Heatmap
ECM remodeling					
Tlr7	1.38	0.00001	-1.44	0.00000	
Lum	1.41	0.43759	-1.45	0.00475	
Mmp1a	-1.04	0.99931	-1.59	0.00000	
Mmp13	3.34	0.00000	-1.89	0.00572	
Mmp14	-1.26	0.19853	-1.48	0.00288	
Cd14	1.26	0.41587	-1.43	0.04289	
Cd44	1.55	0.00061	-1.84	0.00000	
Cd52	1.78	0.00015	-1.72	0.00036	
Cd63	2.03	0.00646	-1.82	0.02941	
Cd68	1.62	0.00095	-1.91	0.00002	
Cd72	1.74	0.00001	-1.47	0.00145	
Cd74	1.86	0.00027	-1.95	0.00010	
Cd84	1.33	0.00656	-1.72	0.00000	
Cd93	1.35	0.00036	-1.44	0.00003	
Cd97	1.22	0.00622	-1.59	0.00000	
Cd207	1.64	0.00825	-1.95	0.00024	
Cd163	-1.29	0.06830	1.58	0.00011	

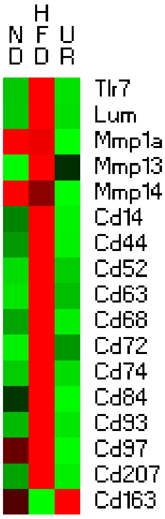

ECM regulation

Col1a1	2.74	0.00001	-1.78	0.01204	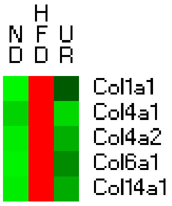
Col4a1	1.66	0.00001	-1.68	0.00001	
Col4a2	1.70	0.00016	-1.52	0.00350	
Col6a1	1.79	0.00032	-1.48	0.02468	
Col14a1	1.62	0.00028	-1.44	0.00641	

Ccl4, chemokine (C-C motif) ligand 4; Ccr5, chemokine (C-C motif) receptor 5; Cxcl, chemokine (C-X-C motif) ligand; Il5ra, interleukin 5 receptor, alpha; Tnfaip2, tumor necrosis factor, alpha-induced protein 2; Tnfrsf, tumor necrosis factor (ligand) superfamily; Adam, a disintegrin and metallopeptidase; Adamts2, a disintegrin-like and metallopeptidase (reprolysin type) with thrombospondin type 1 motif, 2; Casp, caspase; Csf2ra, colony stimulating factor 2 receptor, alpha, low-affinity (granulocyte-macrophage); Saa1, serum amyloid A-1 protein precursor; Ccrn4l, CCR4 carbon catabolite repression 4-like (S. cerevisiae); Il1rap, interleukin 1 receptor accessory protein; Il10rb, interleukin 10 receptor, beta; Tlr7, toll-like receptor 7; Lum, lumican; Mmp, matrix metallopeptidase; Cd, CD antigen; Col1a1, collagen, type I, alpha 1 e; Col4a1, procollagen, type IV, alpha 1; Col4a2, collagen, type IV, alpha 2; Col6a1, procollagen, type VI, alpha 1; Col14a1, collagen, type XIV, alpha 1.

Table S5. Effect of ursolic acid on transcriptional pattern of fibrosis-related genes in muscle of C57BL/6J mice.

Gene	Fold change (HFD/ND)	*p* value	Fold change (UR/HFD)	*p* value	Heatmap
Chemokines and Cytokines					
Adam9	-1.37	0.00191	1.60	0.00001	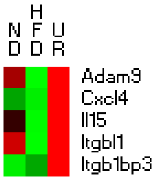
Cxcl4	-1.10	0.59853	1.45	0.00000	
Il15	-1.25	0.03274	1.54	0.00001	
Itgbl1	-1.32	0.14787	1.42	0.02815	
Itgb1bp3	1.16	0.96132	1.53	0.03059	
ECM remodeling					
Dcn	-1.51	0.00032	1.65	0.00002	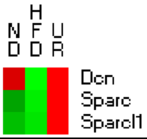
Sparc	-1.19	0.15915	1.97	0.00000	
Sparcl1	-1.06	0.99799	1.49	0.00021	
ECM regulation					
Col1a1	1.02	1.00000	1.65	0.04373	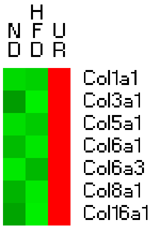
Col3a1	-1.14	0.98834	1.62	0.02670	
Col5a1	1.04	1.00000	1.51	0.01177	
Col6a1	-1.06	0.99814	1.45	0.00076	
Col6a3	1.09	0.99586	1.50	0.01083	
Col8a1	-1.08	0.99757	1.50	0.00644	
Col16a1	-1.09	0.98589	1.42	0.00895	

Adam9, a disintegrin and metallopeptidase domain 9; Cxcl4, chemokine (C-X-C motif) ligand 4; Il15, interleukin 15; Itgbl1, integrin, beta-like 1; Itgb1bp3, integrin beta 1 binding protein 3; Dcn, decorin; Sparc, secreted acidic cysteine rich glycoprotein; Sparcl1, SPARC-like 1 (mast9, hevin); Col1a1, collagen, type I, alpha 1 e; Col3a1, collagen, type III, alpha 1; Col5a1, procollagen, type V, alpha 1; Col6a, procollagen, type VI, alpha; Col8a1, collagen, type VIII, alpha 1; Col16a1, collagen, type XVI, alpha 1. .
